# Supplementary material for: A Novel Variant of the ACTRT1 Gene Is Potentially Associated with Oligoasthenoteratozoospermia, Acrosome Detachment, and Fertilization Failure
Source: Genes (Basel). 2025 Nov 28;16(12):1422. doi: 10.3390/genes16121422 (PMC12733362; doi:10.3390/genes16121422)
Supplement: Supplementary file 1 [file genes-16-01422-s001.zip › genes-4007905-supplementary.pdf]

Supplementary Materials

Table S1. Semen analyses of the patient.

Parameter	Sample 1	Sample 2	Sample 3	Sample 4	Reference Value, WHO 2010
Volume (mL)	2.8	3.1	2.4	3.2	≥1.5
pH	7.6	7.6	7.5	7.5	≥7.2
Sperm concentration (10^6/mL)	2.8	1.2	0.31	0.16	≥15.0
Total sperm count (10^6 per ejaculate)	7.84	3.72	0.74	0.51	≥39.0
Progressive motility, PR (%)	9.0	6.0	6.0	0.0	≥32,0
Total motility (%)	42.0	37.0	51.0	26.0	≥40.0
Morphology (%)	1.0	1.0	0.0	0.0	≥4.0
Vitality (%)	45.0	63.0	91.0	87.0	≥58.0
Head defects (%)	65.0	75.0	68.0	72.0	n.d
Leukocyte concentration (10^6)	0.30	0.04	0.10	0.2	<1.0

n.d.—not defined.

Table S2. TEM results in the patient and a control cohort.

	Intact Sperm Head (%)	Acrosome Hypoplasia (%)	Axoneme Abnormalities (%)	Impaired Chromatin Condensation (%)	Excessive Residual Cytoplasm (%)	Enlarged Subacrosomal Space (%)
**C1**	4	54	9	12	2	2
**C2**	7	57	3	15	5	4
**C3**	4	7	1	9	1	1
**C4**	6	41	10	22	9	6
**C5**	6	61	6	25	11	8
**C6**	4	37	2	14	4	3
**C7**	5	41	16	18	6	5
**C8**	4	59	10	27	10	9
**C9**	7	40	5	7	2	2
**C10**	6	57	24	21	7	7
**C11**	4	53	10	16	5	4
**C12**	5	49	2	26	11	10
**C13**	4	53	7	11	3	3
**C14**	8	43	16	23	8	7
**C15**	9	51	6	17	6	5
**C16**	5	53	17	28	13	11
**C17**	7	47	3	13	3	2
**C18**	4	40	30	24	8	8
**C19**	8	48	9	8	1	1
**C20**	5	47	14	19	7	6
**C21**	4	55	6	15	4	4
**C22**	6	50	3	29	12	10
**C23**	5	43	7	10	1	1
**C24**	5	32	6	20	9	8
**Controls, average ± SD (min-max)**	5.4 ± 1.7 (4-9)	46.0 ± 11.7 (7-61)	9.7 ± 7.6 (1-30)	17.0 ± 8.3 (7-29)	6.2 ± 3.9 (1-13)	5.3 ± 3.4 (1-11)
**Patient**	0	42	31	54	31	49

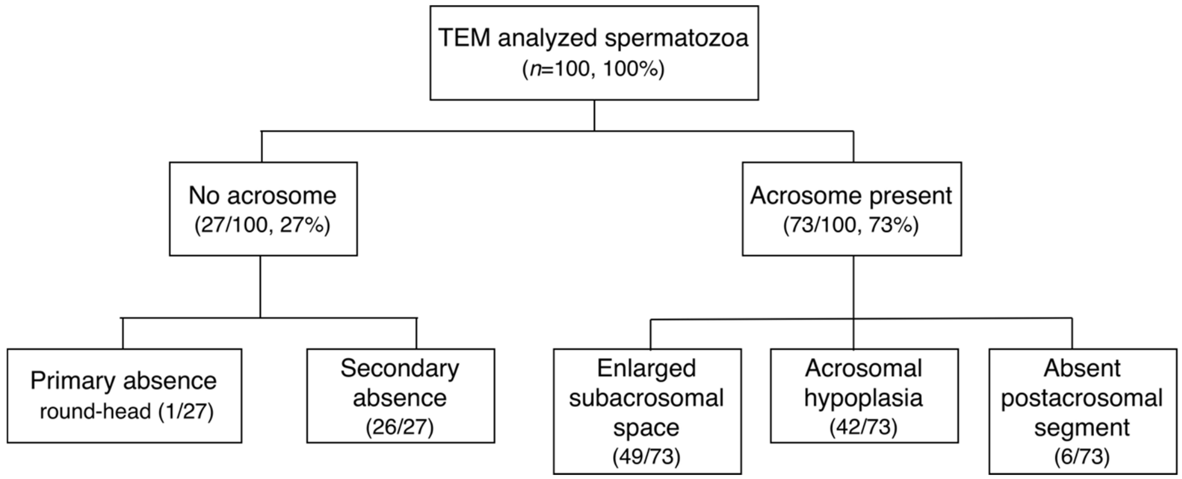

Figure S1. Acrosome abnormalities, detected by TEM.
